# Addressing Adolescents’ Prejudice toward Immigrants: The Role of the Classroom Context

**DOI:** 10.1007/s10964-022-01725-y

**Published:** 2022-12-29

**Authors:** Flavia Albarello, Sara Manganelli, Elisa Cavicchiolo, Fabio Lucidi, Andrea Chirico, Fabio Alivernini

**Affiliations:** 1 Department of Developmental and Social Psychology, Sapienza University of Rome, Rome, Italy; 2 National Institute for the Evaluation of the Education System (INVALSI), Rome, Italy; 3 Department of Systems Medicine, Tor Vergata University of Rome, Rome, Italy

**Keywords:** Attitudes toward immigrants, Stereotype Content model, School, Classroom features, Adolescents, Multilevel analysis

## Abstract

According to social learning theory, classrooms are essential socialization contexts for intergroup attitudes, but analyses of contextual factors net of the impact of individual variables affecting prejudice toward immigrants are very limited. This study was conducted on a large sample of Italian adolescents (*N* = 2904; *M*_age_ = 13.70; females = 48.5%; 168 classrooms). It examined the role of classroom contextual factors affecting adolescents’ prejudice toward immigrants, relying on the combination of groups’ warmth and competence, and their antecedents (i.e., competition and status). Multilevel structural equation analyses revealed that classroom contextual factors (i.e., classroom socio-economic status-SES; classroom open to discussion climate; classroom educational achievements) indirectly affected, at the class level, adolescents’ perceived warmth and competence of immigrants through the mediating role of perceived competition (and status) of immigrants. These findings suggest that interventions targeting the classroom context can help to hinder prejudice in adolescence at the class level.

## Introduction

Prejudice against immigrants is still a critical issue for many Western countries, including Italy (Bergamaschi, 2013; Organization for Economic Co-operation and Development; OECD, 2017). Given that youth is considered a crucial period in life development, comprising becoming more aware of intergroup attitudes and relations (Cavicchiolo et al., 2022) with various social groups (Crocetti et al., 2022), schools represent a core context wherein adolescents can meet others and develop or change attitudes toward various aspects of life (e.g., democratic attitudes; civic participation; Hooghe & Dassonneville, 2013), with important implications for intergroup relations and prejudice (Verkuyten, 2018). Classes – as cultural environments in which adolescents are socialized to the opinions of others and influenced by them (Mitchell, 2019) – play a fundamental role in adolescents’ views about immigrants (Eckstein et al., 2021; Miklikowska et al., 2021), but class-level analyses of the factors affecting adolescents’ prejudice toward immigrant are limited (e.g., Wilson-Daily et al., 2018). The available studies mainly endorsed unidimensional analyses focusing on evaluative dimensions of prejudice toward immigrants depicted as a threat to the ingroup (e.g., “immigrants are troublesome”; “immigrants are annoying”; Alivernini et al., 2019a; “immigrants increase criminality”; van Zalk & Kerr, 2014). To overcome these limitations and increase the knowledge on adolescents’ prejudice, this study endorsed a social psychological account of prejudice toward immigrants neglected in adolescence literature, the stereotype content model (Fiske et al., 2002). Such model allows addressing the specific “contents” of prejudice (i.e., the stereotypical traits attributed to groups; Fiske et al., 2002) targeting a group in terms of the crossing of the two fundamental dimensions of social judgment (i.e., the “Big Two”; Abele & Wojciszke, 2014), namely, *warmth* and *competence*. To the aim of endorsing a thorough understanding of the factors that can explain adolescents’ prejudice in the school context – beyond analyses relying on a partial or a restricted number of factors (e.g., Gniewosz & Noack, 2008) – the study focused on group level effects of multiple contextual factors pertaining to adolescents’ experience in the classroom (i.e., classroom immigrant density and socioeconomic status-SES, classroom open to discussion climate, classroom educational achievement, and classroom civic knowledge) net of the impact of individual variables (Marsh et al., 2012).

### Adolescents and Prejudice toward Immigrants

Social developmental theories of intergroup prejudice stress that children acquire intergroup stereotypes through socialization experiences at a very early age, even before they develop the cognitive skills and flexibility necessary to evaluate such beliefs’ acceptability (Aboud, 1988; Devine, 1989). Only later, due to increased cognitive competencies, prejudice decreases because adolescents move beyond the dichotomous view of “Us versus Them” and have more complex perceptions of their own and others’ identities (e.g., Albarello et al., 2018). Recent meta-analytical evidence confirmed these theoretical assumptions (Crocetti et al., 2021; Raabe & Beelmann, 2011).

Few contributions focused on adolescents’ prejudice and the factors that can shape it (e.g., Eckstein et al., 2021; Miklikowska et al., 2019). Among these, a recent longitudinal study examining the development of prejudice showed that adolescents’ high levels of in-depth exploration of personal educational choices (Albarello & Rubini, 2022a; Crocetti et al., 2008) enhanced individuals’ likelihood of being moderately prejudiced or less prejudiced compared to the risk of being highly prejudiced at a later time (Bobba et al., 2022). This suggests that during adolescence prejudice can still be shaped by several factors, since in this phase individuals experience more complex and differentiated social environment and relationships with others (Sani & Bennett, 2011); they reflect on the position of the ingroup relative to that of outgroups (Verkuyten, 2018), form abstract ideological beliefs (e.g., tolerance), and deeper understanding of moral and egalitarian principles (Rutland & Killen, 2015; van Zalk & Kerr, 2014).

Prejudice against immigrants is a crucial social issue in Italy currently in politicians’ agendas (Bergamaschi, 2013): 8.76% of the total Italian population are immigrants regularly registered in Italy, and in Italian schools, 9.38% of students are immigrants (MIUR-Ministero dell’Istruzione, dell’Università e della Ricerca, 2021). More than 200 nationalities are reported: Romanians are the most numerous immigrant group, followed by Albanians, Moroccans, Chinese, and Ukrainians (Caritas, 2022). The study of (adolescents’) prejudice toward immigrants in Italy is relatively recent (e.g., Albarello et al., 2020; Albarello & Rubini, 2022b): available contributions show that Italians tend to refuse immigrant groups (Alivernini et al., 2019c) perceived as culturally and religiously more different from the ingroup (e.g., Albanians, Moroccans; Kosic et al., 2012; Mancini & Panari, 2010); also economic competition or threat to ingroup’s resources seem to underlie their prejudice toward immigrants (Mancini et al., 2020). Recent research showed that, due to COVID-19 pandemic, prejudice toward highly stigmatized and discriminated outgroups such as immigrants has increased as a reaction to such situational threat (Albarello et al., 2022; Mula et al., 2022). Addressing adolescents’ prejudice toward immigrants in Italy is thus a timely issue that deserves empirical attention.

### A Social Psychological Analysis of Prejudice

Social psychologists have long investigated prejudice as a cornerstone of intergroup relations (e.g., Brown, 2011) to identify its social cognitive roots. Pivotal theorizations stressed the role of *social categorization* (i.e., the distinction between “us” and “them”; Brown, 2011) and motivational processes such as intergroup *competition* over scarce material resources (i.e., the perception that ingroups’ and outgroups’ goals concerning desired resources are in conflict; Scheepers et al., 2002) or the *need to positively distinguish* the ingroup over the outgroup in terms of material but also symbolic (e.g., status, prestige) resources (Brown, 2011). Such general explanations of prejudice cannot account for the different cultural contents of prejudice or stereotypes portraying a group (e.g., Jews are avid; Italians are mobsters; rich people are inhuman and cold; older people are useless; etc.) that do not simply depend on the processes mentioned above. Addressing such limitations, and since many groups do not receive a one-dimensional, hostile type of prejudice (Cuddy et al., 2008), the stereotype content model was formulated to provide a heuristic explanation of the peculiar and different specific contents of prejudice affecting social groups based on the perception of group in terms of *warmth* (i.e., trustworthiness, sociability) and *competence* (i.e., how capable or agentic groups are; Fiske et al., 2002). These two dimensions are psychologically independent (Fiske, 2018). They are universally acknowledged as meaningful dimensions of social perception of others as they help the perceiver to accomplish a fundamental evolutionary pressure (Fiske et al., 2002) – that is, the detection of “friends or foes” (Cuddy et al., 2009, p. 29) – since knowing about a person/group’s warmth and competence allows determining their good or bad intentions, the potential benefits or harms caused by these goals (i.e., the warmth dimension in the model), and their ability to act on those intentions (i.e., the competence dimension in the model).

According to the model, cultural stereotypes/prejudices result from the structural relations between groups so that they serve to justify the status quo (Cuddy et al., 2008). On the one hand, competitive or exploitative groups are stereotyped as lacking warmth, whereas noncompetitive ones are stereotyped as warm (Cuddy et al., 2008). For this reason, perceived competition (which is referred to a group’s intention and behavior) has been theorized as the structural antecedent of warmth, but not of competence (which has not to do with the intention of a group toward the ingroup: a group can be highly competent but at the same time not dangerous for the ingroup; e.g., surgeons are very competent, but usually they are not perceived as having negative intentions toward patients or colleagues). On the other hand, since high-status groups (i.e., having the resources or power to carry out goals) are stereotyped as competent, whereas low-status ones are stereotyped as not competent (Cuddy et al., 2009; Fiske et al., 2002), perceived group’s status has been assumed as the structural antecedent of judgments on groups’ competence, independently of the warmth judgments (e.g., extremely talented piano players might be either warm or cold persons).

This model allows the detection of the multiple dimensions underlying prejudicial portrayals of a group by considering the combination of warmth (high, low) and competence (high, low). As a consequence, four types/clusters of prejudice have been theorized: a) *admiration prejudice* (which is conceived as the most positive pattern of prejudice, usually referred to ingroups) targets highly competent and warm groups who do not compete with other groups; such groups elicit emotional correlates such as pride and admiration and behavioral outcomes such as active and passive facilitation (Cuddy et al., 2008); b) c*ontemptuous prejudice* targets low competence and low warmth groups (e.g., homeless people) that elicit contempt, hate, or disgust, and active and passive harm as a behavioral outcomes; this specific pattern of prejudice is the worst one since – depending on the connection between contempt/disgust and dehumanization (i.e., the denial of full humanness to others; Albarello & Rubini, 2008; Rubini et al., 2017) – it usually conveys a less human perception of the groups (Harris & Fiske, 2006); c) *paternalistic prejudice* targets low competence and high warmth groups (e.g., elderly people), eliciting pity and helping behavior or passive harm; d) *envious prejudice* portrays groups as competent but not warm; that is, they are acknowledged to be doing well (for themselves), but their intentions toward the ingroup are presumed not to be positive (e.g., Asians); passive facilitation and active harm are the behavioral correlates of such form of prejudice.

Even if the stereotype content model has been tested in a comprehensive series of studies, including cross-cultural ones (e.g., Cuddy et al., 2009), developmental studies mainly neglected its application. For instance, it is unknown what the developmental implications of the aforementioned different patterns of prejudice are. Only one study considered whether competence and warmth – and their structural antecedents – underlie adolescents’ perception of two ethnic outgroups (i.e., Moroccans and Ecuadorians; Constantin & Cuadrado, 2020). This contribution revealed unpredicted associations between perceived competition, warmth, and competence depending on the target group, thus suggesting that it is worth investigating the prejudicial perception that adolescents have toward immigrants, since specific stereotype contents might emerge.

### Classroom-Related Antecedents of Adolescents’ Prejudice

Analyzing what happens at school – the domain wherein young people spend most of their time – is crucial (Eccles & Roeser, 2011). Endorsing a social learning perspective (Bandura, 1977), schools can be considered micro-societies allowing adolescents to feel part of society, be included in a democratic environment, and directly experience its consequences on their skin in the context of the classroom (Lenzi et al., 2014); such experiences, in turn, can serve as a template for interaction with others inside and outside school. Looking at the characteristics of the specific classroom context helps unravel the effects of school-related factors on adolescents’ prejudice toward immigrants.

Research has underlined that the school context influences intergroup attitudes considering various factors (e.g., interethnic friendships; Thijs & Verkuyten, 2014). For instance, socialization with peer attitudes has been shown to modify individual’s prejudicial attitudes (Miklikowska et al., 2019). Few studies focused on the classroom level of analysis (e.g., Gniewosz & Noack, 2008): some highlighted the importance of considering individuals’ personal experiences as peculiar and separated from the collective one (e.g., Miklikowska et al., 2019); other contributions underlined that individual perceptions might also vary systematically between students from different classrooms. For instance, class-level average perceptions of a democratic environment, supportive peer relations, and multicultural education are associated with less negative attitudes toward immigrants (Eckstein et al., 2021), meaning that class contexts can display effects on levels of prejudice which cannot be explained based on the individual characteristics of the adolescents.

No study tackled whether adolescents with similar characteristics but attending classrooms with different contexts display anti-immigrant prejudice in terms of the stereotype contents related to groups’ warmth and competence to a different extent (Fiske et al., 2002). Concerning the classroom context, two orders of influential factors can be underlined: background less malleable factors and malleable ones that can somehow be changed by schooling and teachers (Alivernini et al., 2016).

#### Less malleable background factors

Numerous contributions highlighted that high immigrant classroom density was associated with low levels of anti-immigrant prejudice (e.g., Miklikowska et al., 2019), while others underlined more pronounced biases in more diverse classrooms (e.g., Vervoort et al., 2011). If the first evidence is in line with predictions derived from the intergroup contact theory (Pettigrew & Tropp, 2006) stressing that positive intergroup encounters with outgroupers can reduce prejudice toward outgroups, the second one can be explained by complementary theorizations stating that the mixing of different groups may elicit intergroup tensions due to competition over resources, as it is assumed by the realistic group conflict theory or the ethnic competition theory (Scheepers et al., 2002). Given the variability among findings, the effect of immigrant classroom density still needs dedicated attention.

Little evidence is available on the role of SES on anti-immigrant attitudes, and most studies focused on the individual level of analysis. Among these, a negative association between individuals’ objective SES and prejudice has been outlined, showing that higher individual SES slightly corresponds to more positive feelings toward immigrants (Alivernini et al., 2019a) and lesser anti-immigrant prejudice (Gniewosz & Noack, 2008). Such evidence has been explained by stressing that students from more affluent families have more opportunities than their less advantaged peers to visit different countries and understand more about foreign cultures (Alivernini et al., 2019a). As a consequence, on the one hand, this increased contact with different outgroupers (Pettigrew & Tropp, 2006) contributes to broadening adolescents’ views about their society and increases their tolerance toward minorities (Valentine & McDonald 2004); on the other hand, those adolescents who have less access to socioeconomic resources might perceive immigrants as a possible threat to their interests and as competitors for the same resources (Scheepers et al., 2002), thus leading to less positive attitudes toward immigrants.

#### Malleable background factors

Among malleable contextual features, the classroom climate is one of the most commonly studied. It has been shown that adolescents who perceived the classroom climate to be more cooperative had lower levels of anti-immigrant attitudes than those who perceived the classroom climate as less cooperative (Miklikowska et al., 2021). This supports the assumption that (cooperative) classroom experiences act as models (cf. Bandura, 1977) for interethnic relations, reducing prejudice.

Numerous contributions focused on another specific, malleable facet of climate – classroom *openness to discussion* – in which teachers motivate students to feel free to bring up issues to the class, express their own opinions, explore diverse perspectives and respect the opinions of each other (Hahn, 2011). Drawing on the social learning theory (Bandura, 1977), classroom openness to discussion has been conceived as an actualization of democratic values and tolerance (Gniewosz & Noack, 2008), leading to numerous beneficial outcomes: for instance, it is positively associated with more support for human rights, increased levels of political efficacy (Knowles & McCafferty-Wright, 2015) and civic knowledge (Alivernini & Manganelli, 2011), civic engagement (Manganelli et al., 2015) and critical knowledge (Godfrey & Grayman, 2014). In addition, evidence suggests that a classroom climate that is perceived as respectful of students’ different opinions is beneficial to promoting the psychological well-being of immigrant adolescents (Alivernini et al., 2019b). Since in an open to discussion climate, students are also stimulated to elaborate on complex information about political and civic issues (Lin, 2014), they display less negative attitudes toward outgroupers. This has been confirmed in a recent study highlighting that *–* at the classroom level *–* class-average perceptions of a democratic classroom climate, supportive peer relations in class, and multicultural education were associated with less negative attitudes toward immigrants (Eckstein et al., 2021).

Besides climate and direct experiences of tolerance and democratic political attitudes at school, various educational attainments can be analyzed as malleable contextual factors that vary among classes. For instance, available evidence reveals that high educational achievement reduces negative outgroup attitudes (Hjerm et al., 2018); that is, the more a student is educated, the more he/she endorses democratic attitudes toward others (Carrasco et al., 2020; Gniewosz & Noack, 2008). A structural explanation of this association has been provided relying on the ethnic competition theory (Scheepers et al., 2002), according to which, particularly in highly competitive conditions, those with the least resources (i.e., the poorly educated) are more likely to perceive ethnic minorities as a threat than those with more resources (i.e., the well-educated; Hello et al., 2002).

Several contributions reveal that teaching about critical thinking and multiculturalism is negatively associated with anti-immigrant prejudice (Eckstein et al., 2021; Hjerm et al., 2018). Considering specific learnings, *civic knowledge* (i.e., “the knowledge and understanding of civics and citizenship”; Carrasco et al., 2020, p. 192) has been shown to have a relevant role in adolescents’ understanding and reasoning of political issues (Carrasco et al., 2020; Schulz et al., 2008), since it comprises the primary component of citizenship education (Knowles & McCafferty-Wright, 2015), potentially affecting the views that adolescents develop about outgroupers. In particular, it has been shown that adolescents who are low in civic knowledge are less able to handle complex matters, such as evaluating a public policy concerning equality (Shultz et al., 2008); on the contrary, individuals with higher levels of civic knowledge have skills and notions that can help them understand whether public policies can reduce inequalities between social groups. These findings might pair evidence and theorization on the role of increased abstract thinking and skills of adolescents (Kuhn, 2009; e.g., identification with humanity, tolerance; Albarello et al., 2021; van Zalk & Kerr, 2014) in challenging prejudicial attitudes learned in childhood, that is, the more a student develops sophisticated notions in terms of civic knowledge, the lower the level of intergroup prejudice. If existing studies suggest that educational attainments, including civic knowledge, lessen adolescents’ prejudice toward immigrants, their effects as contextual factors at the class level are yet to be explored (Sciffer et al., 2022).

## The Current Study

The study examined the underexplored role of various class-level contextual factors on the specific contents of prejudice displayed by adolescents toward immigrants. As for less malleable background factors, it was expected that adolescents in classes with a higher immigrant density (i.e., having a higher opportunity of contact) would display lesser prejudice toward immigrants expressed in terms of higher warmth and competence (i.e., the most desirable pattern of prejudice) of immigrants than adolescents in classes with lower immigrant density (Hypothesis 1a). Nonetheless, relying on the assumption that immigrants’ presence enhances ethnic competition and prejudice, it could also be expected that classes with high immigrant density would judge immigrants as lower in warmth and competence than classes with low immigrant density (Hypothesis 1b). In the same vein, adolescents in high SES classes would perceive immigrants more positively on warmth and competence than those in low SES classes (Hypothesis 2). As open to discussion climate involves experiencing democratic values, it was expected that an open to discussion classroom climate would be positively associated with more positive judgments of immigrants’ warmth and competence (Hypothesis 3). Given that classes with high achievement might perceive lesser threat/competitiveness by immigrants, it was expected that the higher the classroom educational achievement, the lesser the prejudice toward immigrants expressed as more positive judgments in terms of higher warmth of immigrants and higher competence (Hypothesis 4). Along the same line, classroom civic knowledge was expected to be positively associated with higher judgments of the warmth and competence of immigrants (Hypothesis 5). As for the relation between warmth and competence and their structural antecedents, the perceived competition of immigrants was expected to be negatively associated with their perceived warmth, whereas the perceived status of immigrants would be positively associated with their perceived competence (Hypothesis 6). Overall, the effects of less malleable and malleable contextual classroom features on the warmth and competence of immigrants were expected to be mediated by their two structural antecedents, respectively, perceived competition and status of immigrants (Hypothesis 7).

## Method

### Sample and Procedure

The data analyzed in the present study came from eighth-grade students who participated in the International Civic and Citizenship Education Study 2016 (ICCS 2016; Schulz et al., 2018) in Italy. Grade 8 students were the target population of ICCS: in Italy, this grade represents the last year of lower secondary education and marks the end of the first education cycle. This project was conducted by the International Association for the Evaluation of Educational Achievement (IEA) and aimed to investigate how young people are prepared to undertake their roles as citizens. Participating students were sampled from the whole population of Italian lower secondary schools using the stratified two-stage probability design elaborated by the IEA (Schulz et al., 2018), and they were a nationally representative sample of eighth-grade students. Since this study focused on the prejudice of a majority group toward a salient outgroup (i.e., immigrants), only the responses of the majority group of Italian natives (i.e., the students who had no immigrant background) were examined.

Following the OECD (2017), native adolescents were defined as born in Italy and with at least one parent born in Italy. The final sample of the study included 2873 native adolescents^1^ from 168 classes, their average age was 13.8 years (*SD* = 0.43), and 48.4% were females. The average classroom size was 17.1 students, and the distribution of socioeconomic background was approximately normal (Skewness = 0.29; Kurtosis = −0.51). The data analyzed during the current study are available under request at: https://invalsi-serviziostatistico.cineca.it/.

Data were collected in the classes during an ordinary school day employing the ICCS cognitive test, student questionnaires, and the national option questionnaire, following the IEA assessment protocol (Schulz et al., 2018). Each participating school gave its informed consent, and students were given a standardized introduction, which informed them of the purpose of the study and provided information on how to complete the test and the questionnaires.

### Measures

All variables were measured at the students’ level, except for classroom immigrant density; they were all included in the analysis at both the between-classes level (L2) and within-class level (L1). For each measure, higher scores indicated higher levels of the variable, while lower scores indicated lower levels of the variable.

#### Classroom immigrant density

Classroom immigrant density was measured by computing the proportion of students with an immigrant background in each classroom on the whole number of students in the classroom. Consistently with the definitions of the OECD (2017), students with an immigrant background were defined as those who were either first-generation immigrants (i.e., foreign-born students with foreign-born parents) or second-generation immigrants (i.e., born in Italy with foreign-born parents). Information about the country of birth of the students and their parents were asked to students employing three questions. To compute this variable, the answers from both native and immigrant students from the ICCS original sample (*n* = 3460) were used. Classroom immigrant density ranged from 0 to 0.78, with an average of 0.11 (*SD* = 0.12), and it was the only variable that was analyzed only at the between-classes level.

#### SES

Students’ SES was measured by the National Index of Students’ Socio-Economic Background (Schulz et al., 2018), which was computed from three different indices: the highest occupational status of parents index, the highest educational level of parents index, and the home literacy resources index. The final SES scores were computed by the IEA using the factor scores from a principal component analysis performed on these three indices.

#### Open to discussion classroom climate

A six-item scale assessed students’ perceptions about the presence of a classroom climate open to discussion of political and social issues (Schulz et al., 2018). Students were asked to rate, on a 4-point scale ranging from 1 (*never*) to 4 (*often*), how frequently various events occurred during discussions of political and social issues in the classroom (e.g., “students express opinions in class even when their opinions are different from most of the other students”). The index derived by the IEA from this scale was used in the present study (for a detailed description of the scale and its psychometric properties, please refer to ICCS 2016 Technical report; Schulz et al., 2018).

#### Educational achievement

Students’ educational achievement was measured by averaging their official grades in the Italian language and math, which are expressed as a whole number ranging from 4 to 10. For Italian, school grade includes the knowledge of several aspects of language proficiency (i.e., listening, oral production and interaction, reading and comprehension, writing, vocabulary, and grammar); for math, it includes student’s knowledge and skills in arithmetic, geometry, data analysis, and forecasting. Italian and math are considered the two most important subjects in the Italian school system (Bianchi et al., 2021) and the grades in these subjects are closely related to students’ overall academic achievement measured by standardized achievement tests (Cavicchiolo et al., 2020).

#### Civic knowledge

Civic knowledge was measured using the ICCS 2016 Cognitive Test, consisting of 87 items (78 were multiple-choice, and 9 were constructed-response) which covered two cognitive domains (knowing, reasoning and applying) and four content domains (civic society and systems, civic principles, civic participation, and civic identities). The test items were grouped into 11 clusters, and each student completed one achievement booklet consisting of three of these clusters, according to a balanced rotated design. The Rasch model (Rasch, 1960) and the plausible value methodology were employed to derive the civic knowledge scale. Students’ score on this derived index was used in the present study (for a detailed description of the ICCS 2016 Cognitive Test and its psychometric properties, see ICCS 2016 Technical report; Schulz et al., 2018).

#### Perceived stereotypic traits of immigrants: warmth and competence

The two scales initially developed by Fiske et al. (2002) were used to measure the native adolescents’ perceptions of the stereotypic traits of immigrants along the two dimensions of warmth and competence. Adolescents were asked to evaluate the group of immigrants on five warmth items (e.g., “Immigrants are warm”; “Immigrants are tolerant^2^”) and four competence items (e.g., “Immigrants are competent”; “Immigrants are intelligent^3^^”^), using a 5-point scale from 1 (*strongly disagree*) to 5 (*strongly agree*). These scales have been employed in several studies (e.g., Cuddy et al., 2008, 2009; Fiske et al., 2002, 2007). Cronbach’s alphas in the study were good: 0.88 for warmth and 0.84 for competence.

#### Perceived structural attributes of immigrants: status and competition

Consistently with the stereotype content model (Fiske et al., 2002), the perceived structural attributes of immigrants were the native adolescents’ appraisals of the immigrants’ competitiveness (i.e., competition) and their relative socio-economic status (i.e., status). The two scales developed by Fiske et al. (2002) were used to measure the native adolescents’ perceptions of the structural attributes of immigrants along the two dimensions of competition and status. Adolescents were asked to evaluate the group of immigrants on four competition items (i.e., “Resources that go to immigrants are likely to take away from the resources of Italians”; “The more power immigrants have, the less power Italians are likely to have”; “If immigrants get special breaks, this is likely to make things more difficult for Italians”; “The more rights immigrants have, the less rights Italians are likely to have) and four status items (i.e., “Immigrants are well educated”; Immigrants have prestigious jobs; “Immigrants are economically successful”; “Immigrants have a high cultural level”), using a 5-point scale from 1 (*strongly disagree*) to 5 (*strongly agree*). The scales have already been employed in several studies (e.g., Cuddy et al., 2009; Fiske et al., 2002, 2007). Cronbach’s alphas in the study were good: 0.89 for competition and 0.82 for status.

### Analysis Plan

Consistently with the hierarchical structure of the research questions and data of the present study (students nested within classes), analyses were conducted using the doubly-latent models that integrate multilevel and structural equation modeling approaches (ML-SEM; Marsh et al., 2012). This approach allows the integration of observed and latent variables and separate (and theoretically unbiased) estimation of the effects at each level. Mplus 8.7 (Muthén & Muthén, 2017) with Robust Maximum Likelihood (MLR) estimator was used, taking into consideration two levels: a within-class level (L1) and a between-class level (L2). The fit of the multilevel models was judged by conventional criteria, employing both the MLR chi-square test statistic and fit indices (CFI and RMSEA; Marsh et al., 2012). Missing data in the students’ answers to the questionnaire items ranged from 0.1% to 3.5% and were handled using the Full Information Maximum Likelihood method implemented in Mplus.

In a preliminary phase, a multilevel confirmatory factor analysis (MCFA) was performed to test the measurement models of immigrants’ perceived stereotypical traits and perceived structural attributes. For each of these constructs, a two latent factors structure (i.e., warmth and competence for stereotypes, status and competition for attributes) was posited at both the L1 and L2. All the factor loadings were constrained to be equal between levels (Mehta & Neale, 2005).

A ML-SEM was then performed in order to test the hypotheses. All the variables, except for gender and classroom immigrant density, were standardized and included in the model as having both L1 and L2 variance components. Since the study’s goals aimed at examining the influences of the context on the contents of adolescents’ prejudices toward immigrants, the study focused on the effects at the between-classes level, and used the within-class level effects to control for individual influences. To achieve this aim, between climate and compositional effects based on the characteristics of the variables in the model (Marsh et al., 2012) were distinguished. Climate effects were analyzed for open to discussion classroom climate because it is a classroom climate construct (i.e., the referent of students’ ratings is the classroom). Climate effects are effects of a L2 variable on the corresponding L2 outcome. Instead, compositional effects were analyzed for SES, civic knowledge, educational achievement, and perceived structural attributes of immigrants because they are compositional constructs: the referent of students’ ratings is the individual student, and the L2 construct is an aggregation of these different student characteristics, which is used to describe classroom composition. Compositional effects are the effects of a L2 variable on a L2 outcome, minus the effect of the corresponding L1 variable on the L1 outcome (i.e., the L2 effect after controlling for the corresponding L1 effect).

Consistently with the stereotype content model (Fiske et al., 2002), competition and status were entered into the model as predictors of warmth and competence. The constrained multilevel measurement models examined in the preliminary phase were used in the ML-SEM for these variables. All the other factors were entered into the model as observed variables and predictors of the four dimensions of the stereotype content model. Immigrant classroom density, SES, and gender were entered as exogenous variables, and the model was estimated conditioned on these variables. The effects of all the variables were estimated simultaneously.

## Results

Table 1 presents the correlations, descriptive statistics, and intraclass correlation coefficients (ICC) for the variables under investigation. The results of the MCFAs showed a good fit of the factorial invariance models for perceived stereotypic traits (CFI = 0.974; RMSEA = 0.039) and perceived structural attributes of immigrants (CFI = 0.965; RMSEA = 0.048), thus confirming that these constructs had the same structure at both the between-classes level and within-classes level. All subsequent results are based upon these invariance models.

**Table 1:** Correlations, descriptive statistics and Intra-Class Correlation (ICC) for the study variables

	Variables	1	2	3	4	5	6	7	8	9	10	11	12	13	14	15	16	17	18	19	20	21	22
1	P.I. Warmth Item 1	1																					
2	P.I. Warmth Item 2	0.726^**^	1																				
3	P.I. Warmth Item 3	0.577^**^	0.621^**^	1																			
4	P.I. Warmth Item 4	0.543^**^	0.563^**^	0.536^**^	1																		
5	P.I. Warmth Item 5	0.626^**^	0.647^**^	0.597^**^	0.623^**^	1																	
6	P.I. Competence Item 1	0.491^**^	0.457^**^	0.422^**^	0.444^**^	0.462^**^	1																
7	P.I. Competence Item 2	0.652^**^	0.635^**^	0.552^**^	0.507^**^	0.567^**^	0.557^**^	1															
8	P.I. Competence Item 3	0.577^**^	0.645^**^	0.598^**^	0.493^**^	0.555^**^	0.493^**^	0.656^**^	1														
9	P.I. Competence Item 4	0.560^**^	0.556^**^	0.571^**^	0.519^**^	0.551^**^	0.468^**^	0.650^**^	0.587^**^	1													
10	P.I. Status Item 1	0.220^**^	0.216^**^	0.284^**^	0.242^**^	0.317^**^	0.254^**^	0.231^**^	0.263^**^	0.283^**^	1												
11	P.I. Status Item 2	0.189^**^	0.163^**^	0.236^**^	0.203^**^	0.260^**^	0.232^**^	0.190^**^	0.247^**^	0.242^**^	0.670^**^	1											
12	P.I. Status Item 3	0.339^**^	0.348^**^	0.343^**^	0.327^**^	0.357^**^	0.345^**^	0.372^**^	0.356^**^	0.372^**^	0.451^**^	0.476^**^	1										
13	P.I. Status Item 4	0.297^**^	0.287^**^	0.324^**^	0.290^**^	0.342^**^	0.318^**^	0.316^**^	0.346^**^	0.340^**^	0.474^**^	0.509^**^	0.648^**^	1									
14	P.I. Competition Item 1	−0.188^**^	−0.191^**^	−0.173^**^	−0.182^**^	−0.210^**^	−0.200^**^	−0.187^**^	−0.169^**^	−0.163^**^	0.018	0.030	−0.029	−0.019	1								
15	P.I. Competition Item 2	−0.230^**^	−0.231^**^	−0.243^**^	−0.232^**^	−0.249^**^	−0.241^**^	−0.226^**^	−0.204^**^	−0.201^**^	−0.033	−0.008	−0.089^**^	−0.087^**^	0.661^**^	1							
16	P.I. Competition Item 3	−0.234^**^	−0.267^**^	−0.239^**^	−0.240^**^	−0.276^**^	−0.231^**^	−0.235^**^	−0.225^**^	−0.194^**^	−0.017	−0.012	−0.116^**^	−0.096^**^	0.616^**^	0.751^**^	1						
17	P.I. Competition Item 4	−0.280^**^	−0.290^**^	−0.259^**^	−0.265^**^	−0.279^**^	−0.235^**^	−0.269^**^	−0.246^**^	−0.210^**^	0.019	0.037	−0.093^**^	−0.064^**^	0.528^**^	0.658^**^	0.754^**^	1					
18	Immigrant density	−0.029	−0.058^**^	−0.035	−0.024	−0.020	−0.007	−0.030	−0.039^*^	−0.022	−0.001	0.029	0.019	−0.001	−0.021	0.009	0.025	0.018	1				
19	SES	0.123^**^	0.128^**^	0.096^**^	0.147^**^	0.108^**^	0.117^**^	0.110^**^	0.123^**^	0.068^**^	−0.070^**^	−0.032	0.009	0.006	−0.114^**^	−0.138^**^	−0.151^**^	−0.195^**^	−0.014	1			
20	Open to discussion classroom climate	0.179^**^	0.173^**^	0.150^**^	0.142^**^	0.187^**^	0.156^**^	0.204^**^	0.183^**^	0.161^**^	−0.007	0.018	0.069^**^	0.036	−0.058^**^	−0.073^**^	−0.068^**^	−0.077^**^	−0.089^**^	0.075^**^	1		
21	Civic knowledge	0.209^**^	0.191^**^	0.153^**^	0.203^**^	0.184^**^	0.212^**^	0.200^**^	0.212^**^	0.121^**^	−0.124^**^	−0.065^**^	0.028	−0.015	−0.117^**^	−0.124^**^	−0.134^**^	−0.238^**^	0.047^*^	0.364^**^	0.226^**^	1	
22	Educational Achievement	0.141^**^	0.116^**^	0.120^**^	0.155^**^	0.130^**^	0.143^**^	0.104^**^	0.120^**^	0.071^**^	−0.064^**^	−0.007	0.028	0.024	−0.104^**^	−0.116^**^	−0.123^**^	−0.156^**^	0.018	0.357^**^	0.149^**^	0.507^**^	1
	Mean	3.04	3.09	2.76	2.85	2.79	2.68	3.08	2.89	3.06	1.90	1.80	2.28	2.06	2.51	2.53	2.49	2.13	0.110	0.103	53.531	534.881	7.425
	Standard Deviation	0.943	0.947	0.972	1.010	1.040	0.908	0.908	0.921	0.964	0.876	0.809	0.988	0.864	1.140	1.186	1.244	1.230	0.123	1.010	9.103	81.892	1.146
	Intra-Class Correlation (ICC)	0.099	0.089	0.053	0.064	0.084	0.042	0.085	0.051	0.067	0.047	0.027	0.051	0.051	0.037	0.054	0.059	0.058	–	0.214	0.129	0.179	0.103

*P.I.* Perceived Immigrants

^*^p < 0.05; ^**^p < 0.01

**Fig. 1:**
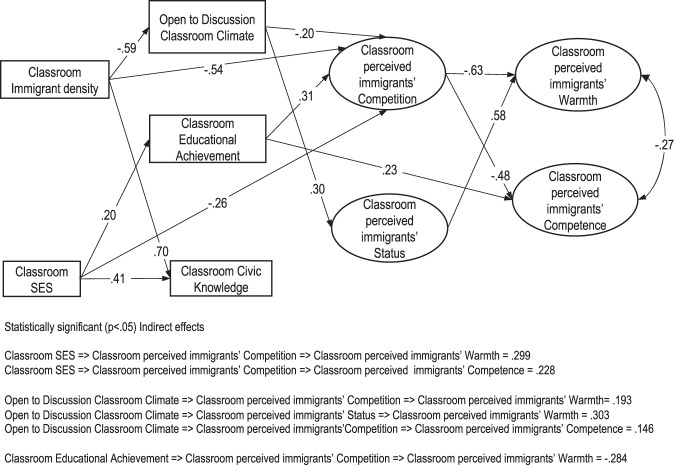
Results of the ML-SEM at the between classes level. Variables were standardized before the analysis. Arrows represent statistically significant relationships (*p* < 0.05)

The results of the multilevel SEM analysis performed to address our hypotheses are summarized in Fig. 1, which shows the results at the between-classes level, taking into consideration the within-class level effects. The fit statistics of the tested model were adequate (CFI = 0.948; RMSEA = 0.034), although the chi-squared was statistically significant (χ^2^_(381)_ = 1647.207; *p* < 0.001), probably because of the large sample size. The model explained almost all the between-classes variance of the perceived stereotypic traits of warmth (91%; *p* < 0.001) and competence (88%; *p* < 0.001) of immigrants and a significant portion of the variance of perceived structural attributes of competition (43%; *p* < 0.05) and status (57%; *p* < 0.001) of immigrants.

As regards the background less malleable factors, results did not support the expectations of the direct effects of immigrant density on immigrants’ perceived warmth and competence (Hypothesis 1a; Hypothesis 1b). Additional findings emerged: higher immigrant density was associated with lower perceived competition, that is, the structural antecedent of warmth. Immigrant density was also positively associated with classroom civic knowledge and negatively with open to discussion classroom climate. Contrary to expectation (Hypothesis 2), after controlling for individual SES, classroom SES was not directly associated with the perceived warmth and competence of immigrants but only negatively with the perceived competition of immigrants at the classroom level. Findings highlighted a further association: classroom SES was positively associated with civic knowledge and educational achievement after controlling for students’ individual SES.

Considering malleable factors, the expected direct effects of open to discussion classroom climate on the two dimensions of prejudice toward immigrants (Hypothesis 3) were not statistically significant. As for the role of classroom educational achievement, the results partially confirmed the expectation (Hypothesis 4) that classroom achievement was positively associated with the perceived competence of immigrants after controlling for students’ individual achievement: equally able students tended to perceive immigrants as more competent when the class-average achievement was high. Classroom achievement was also positively associated with the perceived competition of immigrants after controlling for students’ individual achievement: equally able students tended to perceive immigrants as more competitive when the class-average achievement was high. As expected (Hypothesis 7), through this effect on perceived competition, classroom achievement indirectly influenced the perceived warmth of immigrants, while its indirect effect on perceived competence was not significant. Finally, findings revealed no statistically significant contextual effects of classroom civic knowledge on prejudice toward immigrants (Hypothesis 5) after controlling for students’ individual civic knowledge.

As for the assumptions underlying the relation between warmth and competence and their structural antecedents (Hypothesis 6), findings revealed the expected contextual effects of the perceived competition on the perceived warmth of immigrants: after controlling for students’ individual perceived competition, the class average perception of immigrants’ competition was negatively associated with immigrants’ perceived warmth. Even though unexpected, it was also negatively associated with the perceived competence of immigrants. Native adolescents perceived immigrants as less warm and less competent when they were in classes where immigrants were perceived, on average, as more competitive. There was also a positive contextual effect of the perceived status of immigrants on warmth: native adolescents perceived immigrants as warmer when they were in classes wherein immigrants were perceived as having a high status. Instead, contrary to expectations, there was no statistically significant contextual effect of the perceived status of immigrants on competence.

Finally, the expected indirect effects through classroom perceived competition and status of immigrants on classroom perceived warmth and competence of immigrants (Hypothesis 7) emerged in some specific cases. First, the indirect effect of classroom SES through classroom immigrants’ perceived competition on classroom perceived immigrants’ competence and warmth was significant: in classrooms with high SES, the perceived competition of immigrants at the class level was lower, leading to higher attribution of warmth and competence to immigrants at the class level. Besides this, a significant indirect effect of classroom achievement on classroom perceived competence of immigrants through classroom perceived competition of immigrants emerged. This means that classes with high educational achievements were also classes in which the perceived competition of immigrants was high, leading in turn to lower perceived competence of immigrants at the class level.

The effect of open to discussion classroom climate on perceived immigrants’ warmth was mediated by classroom perceived competition of immigrants: in classrooms with high open to discussion climate, the classroom perception of immigrants’ competition was lower, leading to higher perceived warmth of immigrants at the class level. A significant indirect effect of open to discussion classroom climate on classroom perceived competence of immigrants through class-level perceived competition of immigrants emerged: in classrooms with high open to discussion climate, lower competition was attributed to immigrants at the class level, leading to higher classroom attribution of competence to immigrants. Classroom perceived status mediated the effect of open to discussion classroom climate on classroom perceived warmth of immigrants: classes with a high open to discussion climate were also classes with a higher class-level perception of immigrants’ status, leading in turn to higher attribution of warmth to immigrants at the class level.

## Discussion

Schools and classes are considered socialization contexts for adolescents’ views about others, but class-level analyses on the role of contextual school-related factors are very limited (Mitchell, 2019), and studies in the Italian context are almost missing. Notably, most studies addressed adolescent’s prejudice toward immigrants in terms of unidimensional attitudinal measures (Eckstein et al., 2021; Van Zalk & Kerr, 2014). This study aimed to tackle these gaps by applying a neglected model in the literature on adolescents’ prejudice (i.e., the stereotype content model) to highlight the specific contents of adolescents’ prejudice toward immigrants in terms of warmth and competence, the two fundamental dimensions of social judgment (Abele & Wojciszke, 2014; Fiske et al., 2002). This study employed a large and representative sample of Italian adolescents. By focusing on the effects of classroom-related factors over and above what can be explained by individual features, it underlined how the contents of adolescents’ prejudice toward immigrants were related to different types of contextual antecedents, disentangling the role of background given factors and of malleable school-related factors. Overall, the evidence of this study suggests that – by combining an educational and social-psychological analysis of class-related processes – the knowledge of antecedents of adolescents’ prejudice toward immigrants can be advanced.

### The Contents of Adolescents’ Prejudice toward Immigrants Depending on School-Related Contextual Factors

Compared to the limited available evidence, the study provided a more thorough analysis of factors that can affect native adolescents’ prejudice toward immigrants and lead to more or less desirable class-level evaluations of such outgroup in terms of perceived warmth and competence. Background less malleable factors related to the composition of classes did not directly affect perceived warmth or competence (Hypothesis 1a; Hypothesis 1b; Hypothesis 2). Immigrant density was negatively associated with the perceived competition of immigrants. In line with the intergroup contact theory (Pettigrew & Tropp, 2006), the perceived competitiveness of immigrants was lower in classes with high immigrant density. In this vein, findings seem to provide more support for the beneficial role of contact with immigrants (Tropp & Pettigrew, 2005) rather than the complementary theorization stressing that the higher the presence of immigrants, the higher the natives’ perception of immigrants’ threat and competition for resources (Esses et al., 2001; Scheepers et al., 2002).

Besides this, on the one side, higher ethnic diversity in the classroom also emerged as a facilitator of class-level achievement in civic knowledge, pairing evidence that more socio-economically diverse classes develop higher civic knowledge (Collado et al., 2015). On the other side, it worked as an obstacle to the classroom perceived and experienced democratic functioning (i.e., classroom open to discussion climate). This finding adds to available evidence mainly considering open to discussion as a moderator of the effects of classroom diversity on youth prejudice (e.g., Miklikowska et al., 2021) by showing that also immigrant density might limit school-related outcomes.

As for classroom SES, classes with low SES perceived immigrants as more competitive than classes with high SES. In turn, and in line with expectations (Hypothesis 7), competitiveness mediated the effect of classroom SES on judgments of immigrants’ warmth and competence: if classes with higher SES also perceived lower immigrants’ competitiveness, leading to better classroom evaluation of immigrants in terms of warmth and competence, low classroom SES was associated with more downward attribution *–* at the class level *–* of warmth and competence to immigrants through higher perceived competitiveness. Such evidence can be conceived as coherent with theorizations stressing that natives can perceive immigrants as a threat to the ingroup’s welfare (e.g., Scheepers et al., 2002; Stephan & Stephan, 2000), representing *resource stress* (i.e., the perception that within a society, access to desired resources is limited) that leads to perceived group competition for resources (Esses et al., 2001). In this vein, native adolescents in classes with low SES perceive higher stress regarding access to resources due to immigrants, a very salient outgroup in one’s social environment (Bergamaschi, 2013). This, in turn, leads to attributing lesser warmth and competence to immigrants. Also a direct association between classroom SES and more malleable contextual features such as classroom civic knowledge and educational achievement emerged. The attainment gap depending on low SES is confirmed by the findings that classes with high SES also had better educational achievement (for a review, see Sirin, 2005) and civic knowledge attainments (Collado et al., 2015).

Overall, and most importantly, the evidence mentioned above widens the understanding of the role that such background, less malleable factors related to classroom compositions have on a specific antecedent of prejudice toward immigrants: their perceived competitiveness. Low immigrant density and low classroom SES emerged as factors that can promote a competitive view of immigrants at the class level. This is a crucial point since warmth traits are also shown to have a primacy in person/others’ perception (Cuddy et al., 2009) because, from an evolutionary account, there are potentially greater costs for dealing with someone who is not warm versus not competent (Cuddy et al., 2009; Wojciszke, 2005). This, in turn, might be predictive of the potential behavioral correlates of prejudice, as theorized in the stereotype content model, in terms of active behaviors, both harmful and facilitative, that can target immigrants (Cuddy et al., 2009).

By adding to the minimal evidence on the stereotype content model in the domain of adolescents’ prejudice, this study points to the critical role of experiencing a classroom democratic climate in shaping class-level perceptions of immigrants’ warmth and competence. In particular, even if the predicted direct effect of open to discussion classroom climate on immigrants’ warmth and competence was not found, evidence revealed an indirect effect at the class level through the mediating role of perceived competition and status (Hypothesis 7). Compared to classes with a high open to discussion climate, low classroom open to discussion enhanced the perceived competitiveness of immigrants *–* leading to lower class-level ratings in terms of warmth and competence – and led to the attribution of lower social status *–* leading to lower perceived warmth of immigrants. Interestingly – even if it is not in line with the assumptions of the stereotype content model *–* attributions of status to immigrants were not associated with class-level judgments of immigrants’ competence.

These findings suggest that actual experiences in contexts of democratic and tolerant interactions can be transferred to the views that adolescents develop regarding immigrants (Bandura, 1977; Eckstein et al., 2021). Those who experience less democratic and participative classroom environments are exposed to risk of perceiving higher competitiveness by immigrants and attributing them lower social status, thus perceiving immigrants as lower in warmth and competence. This corresponds to an increased tendency toward a contemptuous-like pattern of prejudice at the class level. Those who experience democratic values and interactions on their own skin are more prone to attribute higher status and low competitiveness to immigrant attributing them higher warmth and higher competence, and displaying a tendency toward an increase in the admiration-like pattern of prejudice (i.e., the most desirable one) that is reserved for the ingroup or admired groups.

The unexpected finding (related to Hypothesis 6) that classroom perceived status of immigrants was not associated with their perceived competence, but with their warmth should not be conceived as a limitation to replication of the stereotype content model (Fiske et al., 2002) given that this was the first time that immigrants’ perception was treated at the group level of shared representations, a very specific context. The analytical approach of the study highlighted the group processes that might act at the class level by controlling for individual perceptions. Thus, it can be argued that in contexts/classes where the perception of immigrants’ competitiveness was high, the acknowledgment of their competence was driven by competition rather than status, leading to lower judgments in terms of warmth and competence than in classes with low perceived competition. Instead, when adolescents were exposed to a class-level perception of immigrants as having high status, the perception of their warmth was also driven by their perceived status.

Also, classroom achievement emerged as a factor affecting adolescents’ prejudice toward immigrants. In particular, classes with high achievements attributed higher competence to immigrants (Hypothesis 4), but not warmth. Educational achievement indirectly affected immigrants’ perceived warmth and competence through the enhanced perception of immigrants’ competitiveness. These findings can be interpreted with the lens of the social identity approach of intragroup and intergroup relations (Ellemers et al., 2002; Sani & Bennett, 2011). In classes with high mean achievements individuals might have difficulties obtaining a positive self-image if they rely on interpersonal, intragroup comparisons within the class, given that the average of students has high academic performances. Consequently, they might enhance the salience of intergroup comparisons and set ingroup-favorable intergroup differentiation by perceiving the outgroup as more competitive and, in turn, less warm. This result can be better understood considering the so-called big-fish-little-pond phenomenon. Evidence of this effect stresses that individual self-concept is weaker in classes with high average achievement (Marsh et al., 2012). Thus, in such classes, intergroup comparisons might become more salient in order to restore self-esteem through ingroup’s evaluation (Brown, 2011; Ellemers et al., 2002).

Finally, classroom civic knowledge seemed to be unrelated to stereotypical classroom perceptions of immigrants in terms of warmth and competence. That is, students with equal levels of civic knowledge tended to have the same prejudices toward immigrants even if they were in classes with very different average levels of civic knowledge. This suggests that formal knowledge about society’s functioning does not relate to prejudices, which are social cognitive products of social interaction (Ellemers et al., 2002) that mere cognition or knowledge seems not strong enough to challenge, at least at the class level. This result matches those observed in a previous study in which civic knowledge alone was insufficient to promote adolescents’ civic engagement (Manganelli et al., 2014).

### Limitations and Future Directions

This study consisted of a large-scale survey conducted with native Italian adolescents. In this vein, it did not consider local differences in immigrants’ presence outside school (besides interethnic classroom composition) and the different facets of contact with immigrants (e.g., direct or extended; positive or negative; Tropp & Pettigrew, 2005). Even though the study’s evidence stressed a positive association between high immigrant classroom density and low perceived immigrants’ competition, future contributions should tackle more closely the quality (either positive or negative) of contact to clarify controversial evidence of contact effects in schools (e.g., Vervoort et al., 2011).

Moreover, in order to get a clear picture of how school-related contextual factors affect adolescents’ prejudice toward a salient outgroup, the study focused only on the majority group of natives; thus, it could not describe the antecedents of minority adolescents’ prejudice toward the majority outgroup (Tropp & Pettigrew, 2005). Besides this, the study endorsed a social identity approach to explain how class-related phenomena/features affect prejudice, relying on the assumption that classes are social groups and are subjected to the same processes explaining interpersonal and intergroup relations (Ellemers et al., 2002). Nonetheless, future studies are needed to directly tackle the motivational bases (e.g., the need to differentiate the ingroup and the outgroup positively) of prejudice toward immigrants displayed by adolescents in classes with high mean achievements.

A further step toward thoroughly understanding the processes leading adolescents to show specific patterns of prejudice toward the outgroup of immigrants could be considering the adolescents’ evaluation of their ingroup (i.e., natives) since it has been consistently shown that compensation effects appear: if a group is attributed one of the two fundamental dimensions of social judgment, the other group involved in the social comparison process would be attributed mainly the other dimension (Yzerbyt et al., 2008). Also, the expected behavioral correlates of the patterns of prejudice could be directly examined (Cuddy et al., 2009).

If the study’s main finding is that malleable school-related classroom features can affect the shared representations that classes hold about immigrants – which is a finding that has field relevance – implementation strategies to achieve this goal have to be designed. A more thorough analysis of why civic knowledge did not affect the class-level contents of prejudice in terms of groups’ warmth and competence is needed. Future studies should also test the effectiveness of interventions aimed at changing the classroom democratic climate and achievements to change adolescents’ prejudice and promote their harmonious intergroup relations with minority outgroups later in life.

## Conclusion

Class-level analyses on the role of contextual school-related factors on adolescents’ prejudice are limited, and most studies focused on unidimensional attitudinal measures of anti-immigrant attitudes. Moreover, prejudice against immigrants is still a critical issue for many Western countries, and studies that examine factors that can be responsible for this prejudice are needed. The current study tackled these gaps by applying a neglected and underexplored model in the literature on adolescents’ prejudice (i.e., the stereotype content model) and focusing on the effects of multiple classroom-related factors over and above what can be explained by individual features. In contrast to evidence and theorizations stressing that prejudice does not change in adolescence, this study suggests that adolescents’ prejudice can be changed. Focusing on group-level processes, malleable and less malleable classroom background factors (i.e., classroom SES, classroom open to discussion climate classroom, and educational achievement), via the mediation of perceived competition and status of immigrants, can mold the pattern of prejudice that young people display toward them: some appear to lower the perceived warmth and competence of immigrants, thus enhancing the risk of showing contemptuous-like prejudice (e.g., low classroom open to discussion climate; low classroom SES); some enhance the perceived warmth and competence of immigrants, thus fostering admiration-like prejudice (e.g., high classroom open to discussion climate, high classroom SES). By combining social-psychological theorizations with insights from educational psychology, the study fruitfully helped to understand more deeply prejudice in adolescence, suggesting that what happens within classes can be profitably manipulated and used to challenge adolescents’ prejudice toward immigrants.

## Footnotes

1 Only majority students were selected in order to obtain a clearer picture of Italian adolescents’ prejudice toward the general outgroup of immigrants, who represent a minority group in Italy (ISTAT, 2022). Since theorization and research on intergroup contact effects have shown that the effect of contact with the outgroup varies depending on whether respondents belong to the majority or the minority groups (e.g., Tropp & Pettigrew, 2005), also the effect of classroom immigrant density (that might be considered as an indicator of intergroup contact) might differ across these two subgroups. For this reason, minority adolescents were not considered in the sample.

2 As can be derived from the stereotype content model, the trait “tolerant” is used to asses a group’s warmth, since it reflects others’ perceived intent in the social context, underlining that “the possessor’s intentions are not harmful for the self or the ingroup” (cf. Cuddy et al., 2008, p. 63).

3 In line with the stereotype content model’s definition of competence, the trait “intelligent” is intended to reflect the “possessor’s chance of achieving personal goals” (Fiske et al., 2007, p. 79).
